# Ventricular Netrin-1 deficiency leads to defective pyramidal decussation and mirror movement in mice

**DOI:** 10.1038/s41419-024-06719-1

**Published:** 2024-05-17

**Authors:** Ling Hu, Xi-Yue Liu, Li Zhao, Zhi-Bin Hu, Ze-Xuan Li, Wei-Tang Liu, Ning-Ning Song, Yun-Qing Hu, Luo-Peng Jiang, Lei Zhang, Yun-Chao Tao, Qiong Zhang, Jia-Yin Chen, Bing Lang, Yu-Bing Wang, Lei Yue, Yu-Qiang Ding

**Affiliations:** 1 Department of Laboratory Animal Science, Fudan University, Shanghai, 200032 China; 2 State Key Laboratory of Medical Neurobiology and MOE Frontiers Center for Brain Science, Institutes of Brain Science, Fudan University, Shanghai, 200032 China; 3 Key Laboratory of Arrhythmias, Ministry of Education, East Hospital, and Department of Anatomy and Neurobiology, Tongji University School of Medicine, Shanghai, 200092 China; 4 Department of Psychiatry, The Second Xiangya Hospital, Central South University, Changsha, 410083 China; 5 Shanghai Institute of Infectious Disease and Biosecurity, Fudan University, Shanghai, 200032 China

**Keywords:** Disease model, Diseases of the nervous system

## Abstract

The corticospinal tract (CST) is the principal neural pathway responsible for conducting voluntary movement in the vertebrate nervous system. Netrin-1 is a well-known guidance molecule for midline crossing of commissural axons during embryonic development. Families with inherited Netrin-1 mutations display congenital mirror movements (CMM), which are associated with malformations of pyramidal decussation in most cases. Here, we investigated the role of Netrin-1 in CST formation by generating conditional knockout (CKO) mice using a Gfap-driven Cre line. A large proportion of CST axons spread laterally in the ventral medulla oblongata, failed to decussate and descended in the ipsilateral spinal white matter of Ntn1^Gfap^ CKO mice. *Netrin-1* mRNA was expressed in the ventral ventricular zone (VZ) and midline, while Netrin-1 protein was transported by radial glial cells to the ventral medulla, through which CST axons pass. The level of transported Netrin-1 protein was significantly reduced in Ntn1^Gfap^ CKO mice. In addition, Ntn1^Gfap^ CKO mice displayed increased symmetric movements. Our findings indicate that VZ-derived Netrin-1 deletion leads to an abnormal trajectory of the CST in the spinal cord due to the failure of CST midline crossing and provides novel evidence supporting the idea that the Netrin-1 signalling pathway is involved in the pathogenesis of CMM.

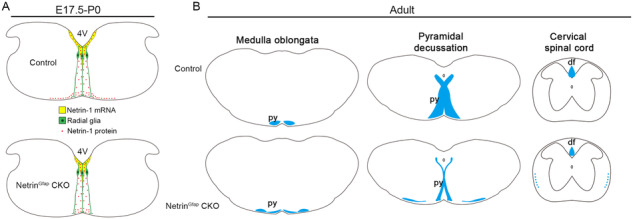

## Introduction

Corticospinal tract (CST) neurons are pyramidal neurons located in the motor and associated cortices, and their axons condense into fiber bundles that travel long distances from the cortex to the contralateral spinal cord to conduct voluntary movements. The axons cross the midline at the junction between the medulla oblongata and the spinal cord, forming a pyramidal decussation and eventually entering the dorsal funiculus of the contralateral spinal cord [1, 2]. The CST trajectory involves successive choice points during embryonic and postnatal development, each of which is guided by a different set of molecular cues [3]. Developmental abnormalities in the CST are associated with the pathogenesis of congenital mirror movements (CMM), a disorder characterized by involuntary symmetrical movements of one hand that mirror intentional movements of the other hand. Malformations of pyramidal decussation CMM are involved in most cases of CMM [4, 5].

Netrin-1 is expressed in the ventral ventricular zone (VZ) and floor plate of the neural tube and binds to several transmembrane receptors, including deleted in colorectal carcinoma (DCC) [6] and Uuc5 [7], functioning as a key guidance factor in promoting axonal growth and mediating the midline crossing of the commissural axons [8, 9]. Netrin-1-knockout mice exhibit defects in the corpus callosum, anterior commissure, hippocampal commissure, and commissural axons of the spinal cord [10, 11]. In human, inherited mutations in Netrin-1 have been identified in two unrelated families with CMM that display abnormal anatomy of the CST [12]. Mutations in DCC have also been shown to cause CMM [13, 14]. These findings strongly imply the role of Netrin-1 signaling in CST axon pathfinding, which has been explored in multiple mouse models. In Kanga mice carrying a spontaneous DCC mutation lacking the exon encoding the P3 intracellular domain, CST axons do not cross the midline but form two bundles that remain in the ipsilateral ventral spinal cord [15]. Moreover, in the absence of Unc5c, the CST splits into two bundles with different trajectories: the lateral bundle does not cross the midline and descends into the lateral white matter of the spinal cord, whereas the medial bundle crosses the midline and reaches the dorsal gray matter. Netrin-1 mutants show reduced pyramidal decussation on postnatal day 0 (P0) [15], the time point at which CST axons initiate midline crossing [16]. However, the neonatal lethality of Netrin-1 mutants makes it impossible to investigate whether Netrin-1 deletion affects the CST trajectory in the spinal cord of adult mice. A recent study has shown that the expression of Netrin-1 in the floor plate of hindbrain is involved in the midline crossing of CST axons [17]. The aim of the present study is to determine whether Netrin-1 expression in the VZ of hindbrain and the cerebral cortex, where the CST neurons are located is required for the development of CST focusing on the decussation process and its trajectory in the spinal cord. In order to achieve the Netrin deletion specifically in the VZ of hindbrain, we employed Gfap-Cre mice, in which the onset of Cre expressing in the VZ begins by E13.5 without Cre activity in the floor plate [18].

In Ntn1^Gfap^ CKO mice, a large proportion of axons spread laterally in the ventral medulla oblongata, failed to decussate, and descended into the ipsilateral spinal white matter. On the other hand, the data from forebrain-specific CKO mice using Emx1-Cre showed that the CST axons were well-maintained. Distinct localizations of Netrin-1 mRNA and protein were found in the embryonic hindbrain, and Netrin-1 protein was distributed in the ventral margin through which the CST axons pass in control mice, but it was largely diminished in Ntn1^Gfap^ CKO mice. Consequently, symmetric forelimb movement was observed in CKO mice. Our results indicated that deletion of Netrin-1 in the VZ of the hindbrain leads to abnormal location of the CST in the spinal cord due to the failure of the CST midline crossing, thus adding novel evidence supporting the idea that the Netrin-1 signalling pathway is involved in the pathogenesis of CMM.

## Materials and methods

### Experimental animals

Ntn1^flox/flox^ mice were generated by inserting LoxP sites flanking exon 2 (Nanjing Jicui Co. Ltd., China) and crossing with Gfap-Cre and Emx1-Cre mice to obtain Ntn1^Gfap^ CKO (Gfap-Cre: Ntn1^flox/flox^) and Ntn1^Emx1^ CKO (Emx1-Cre: Ntn1^flox/flox^) mice, respectively. Littermates of other genotypes (e.g., Ntn1^flox/+^ and Ntn1^flox/flox^) were used as controls. All procedures were performed in compliance with the Animal Experimental Ethics Committee of Shanghai Medical School, Fudan University, China.

### Western blot

Total proteins from the desired regions were prepared and subjected to western blot as described previously [19]. Equal amounts of boiled proteins were fractionated using sodium dodecyl sulfate-polyacrylamide gel electrophoresis and transferred to nitrocellulose membranes. Membranes were then incubated overnight at 4 °C with the following primary antibodies: rabbit anti-Netrin-1 (1:1000, ab126729, Abcam) and rabbit anti-GAPDH (1:1000, LF206, Epizyme), followed by incubation with HRP-conjugated goat anti-rabbit (1:1000; KangCheng, China) for 2 hours at room temperature. Signals were then subjected to chemiluminescence detection (Thermo Scientific).

### Immunostaining, AuCl_3_ staining, and in situ hybridization

Adult mice were anesthetized with sodium pentobarbital (80 mg/kg) and perfused with 4% paraformaldehyde (PFA). Brains were dissected, post-fixed in 4% PFA overnight, cryoprotected in 30% sucrose for 2 days, and cut into 20-µm-thick sections. For immunohistochemical analysis, brain sections were incubated with primary antibodies at 4 °C overnight, and then incubated with biotin-conjugated secondary antibodies (1:500, Jackson ImmunoResearch) at room temperature for 3 hours, which were followed by incubation with streptavidin-Cy3 (1:1000, Jackson ImmunoResearch) and counterstaining with Hoechst 33258 (1:2000, Sigma) at room temperature for 1 hour. Images were acquired using the Eclipse fluorescence microscope (Nikon, Tokyo, Japan). The primary antibodies were listed as follows: rabbit anti-Netrin-1 (1:200, ab126729, Abcam), rabbit anti-protein kinase C gamma (PKCγ, 1:300, sc-211, Santa Cruz), and mouse anti-NFM (1:100; 2H3, DSHB), rat anti-Ctip2 (1:300, ab18465, Abcam) and rabbit anti-Glast (1:300, ab416, Abcam).

AuCl_3_ staining was performed as described previously [20]. Brain sections were stained with 0.2% gold chloride (AuCl_3_) in 0.1 M phosphate-buffered saline. Once axonal staining was evident, the sections were transferred to anhydrous 2.5% sodium thiosulfate for 5 min to terminate the reaction. Notably, this reaction occurred in the dark. For Nissl staining, brain sections were incubated in 1% crystal violet for 1 hour, then sequentially washed with 80% ethanol, 95% ethanol, 100% ethanol and 100% ethanol for 5 min each, performed as previously described [21].

An in situ probe specific to exon 2 of Netrin-1 was generated, and hybridization was performed as described in our previous study [22]. The RNA probes of *PlxnD1* and *DKK3* were constructed according to the Allen Brain Atlas.

For fluorescence in situ hybridization and immunostaining, 10 μg/ml protease K and anti-Digoxigenin-AP (1:1000, 11277073910, Roche) were replaced with 2 μg/ml and anti-Digoxigenin-POD (1:100, 11207733910, Roche), respectively. At the final stage. TSA-FITC was employed to visualize the signal of mRNA followed by the procedures of immunostaining with goat anti-GFP (1:1000, NBP100-1770, Novus Biologicals).

### Tracing of the CST

Adult mice were anesthetized with sodium pentobarbital and injected with 10% biotinylated dextran amine (BDA; MW10000, Invitrogen) into the right motor cortex. Six 0.2-μl aliquots were injected (0.1 μl/min) with a stereotaxic apparatus at the following coordinates: (i) A (anteriority) = 1, L (laterality) = 2, D (depth) = 1; (ii) A = 1, L = 1, D = 1; (iii) A = − 0.25, L = 2, D = 1; (iv) A = − 0.25, L = 1, D = 1; (v) A = − 1, L = 2, D = 1; and (vi) A= − 1, L = 1, D = 1. At each injection point, the needle was left in place for 5 min to minimize leakage.

Two weeks later, the mice were deeply anesthetized and perfused with 4% PFA. Brain slices were prepared as described above, incubated with streptavidin-Cy3 (1:1000; Jackson ImmunoResearch) and counterstained with Hoechst 33258 (1:2000; Sigma) to visualize BDA labeling.

### Behavioral tests

Adult (4–6 months old) male and female mice were used in the following tests. All behavioral experiments were conducted during the light phase of the light/dark cycle in a soundproof room. The mice were habituated for at least 30 min before testing. The experimenter was blinded to the group identities of tested mice. The mice in each group were randomly selected according to the genotyping.

#### Open-field test

The open-field apparatus was a transparent plexiglass box consisting of a square arena with a white floor divided into nine squares. The mice were allowed to explore freely for 30 min, and the total distance was recorded using Activity Monitor software (Med Associates, St. Albans, VT, United States).

#### Rotarod test

In the rotarod test, mice were trained to stay on the rotating rod at a constant speed for a minimum of 1 min and tested over a total of three trials with an accelerating velocity, which started from 3.5 rpm/s to 40 rpm/s by 0.2 rpm/s increments. The latency to fall was reported.

#### The pole test

This test was conducted with a 50-cm-high, gauze-taped pole (1 cm in diameter). The mice were placed on the pole, and the total time until the animal descended to the floor was recorded [23]. Data were recorded thrice and averaged.

#### Reaching exploratory behavior

When placed in a new glass cylinder, the mice tended to contact the walls with their forepaws, which was performed with two paws simultaneously (symmetric movement) or alternatively (asymmetric movement). Ten reaching movements were recorded, and the percentage of symmetric movements was calculated [24].

#### Catwalk

The CatWalk XT (Noldus Information Technology) system included a 1.0-m enclosed walkway. The mice traversed from one side to the other, and recordings were made. Only runs in which the mice passed within 5 s were retained. Each individual footprint was digitalized, and numerous parameters were quantified using CatWalk XT 10.6.608 software [25].

### Statistical analysis

Statistical analysis were performed using GraphPad Prism 8.0 software. All data were presented as mean ± standard error of the mean (SEM) and analyzed using Student’s *t* test. Results were considered significant when the *p* value was <0.05. The number of samples represented biological replicates and was indicated in the figure legends. The area of positive PKCγ in dorsal funiculus was measured from three sections randomly selected from three different animals.

## Results

### Defective pyramidal decussation in adult Ntn1^Gfap^ CKO mice

**Fig. 1:**
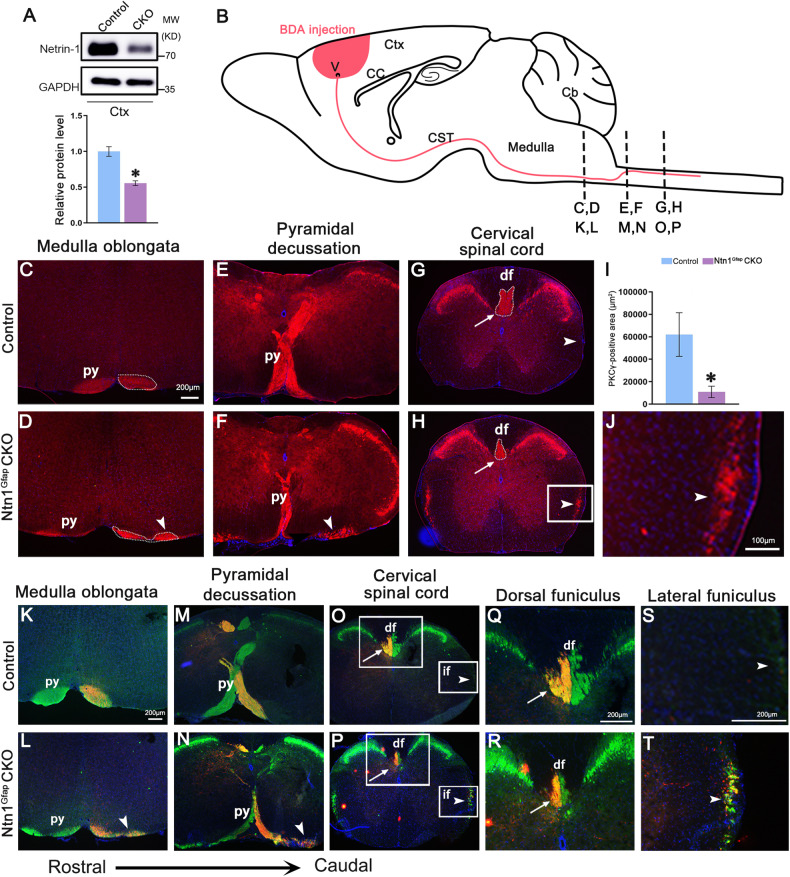
Abnormal trajectory of the CST in Ntn1^Gfap^ CKO mice. **A** Western blot shows the decreased expression of Netrin-1 in the cortex of CKO mice. By Shapiro–Wilk test, the samples followed a normal distribution. Student’s *t* test, *p* < 0.05, *N = 3 for each. **B** Schematic representation of a sagittal section indicating the trajectory of the CST and the level of the coronal sections presented in this study. **C**–**J** Staining with anti-PKCγ antibody shows that the CST axons spread into two bundles, the medial and lateral one (arrowhead), in the ventral hindbrain of CKO mice (**C**, **D**). Besides, pyramidal decussation is much decreased (**E**, **F**), and PKCγ-stained area in the dorsal funiculus (arrow) is also reduced in CKO mice compared with that in control mice (**G**–**J**), **J** is enlargement of the boxed area in **H**. Arrowhead in **H** point to ectopic CST axons in the lateral funiculus of the CKO mice. **I** is quantification of PKCγ-stained area in the dorsal funiculus. By Shapiro–Wilk test, the samples followed a normal distribution. Student’s *t* test, *p* < 0.05, *N = 4 for each. **K**–**T** BDA is unilaterally injected into the right motor cortex and the CST is visualized by both PKCγ immunostaining (green) and BDA (red), showing the split pyramidal tract in the ventral hindbrain (arrowhead in L, N), reduced pyramidal decussation, and reduction of crossed CST axons in the dorsal funiculus (arrow), and uncrossed CST axons in the lateral funiculus (arrowhead in **P**, **T**) of CKO mice. This experiment was replicated for three times. Boxed areas in **O**, **P** are enlarged in **Q**, **S**, and **R**, **T**, respectively. Scale bars = 100 μm in **J**, and 200 μm for the other panels. Cb cerebellum, cc corpus callosum, CST corticospinal tract, Ctx cerebral cortex, df dorsal funiculus, if lateral funiculus, py pyramidal tract.

To examine whether morphological alterations of the CST were present in adult mice with central deletion of Netrin-1, we generated Ntn1^Gfap^ CKO mice (Fig. 1A), which survived postnatally to adulthood with a normal appearance compared with control mice. We then investigated the location of CST axons by immunostaining with PKCγ, a reliable marker of CST axons in mice [26]. CST axons formed the internal capsule while passing through the striatum and diencephalon and served as the major component of the cerebral peduncle at the level of the midbrain. No apparent differences were observed between controls and Ntn1^Gfap^ CKO mice in terms of the location and amounts of axons (Fig. S1). Whereas in the route to the pyramidal decussation, PKCγ-stained pyramidal tract was evident in the ventral hindbrain of control mice, but it broadened in the mediolateral direction and spread into two bundles in the CKO mice: the medial bundle was maintained in the normal position, but the other one was located laterally (Fig. 1B–J, Fig. S1). At the level of pyramidal decussation, labeled CST axons turned dorsally and crossed the midline in control mice. However, in Ntn1^Gfap^ CKO mice, decussation was significantly reduced because the laterally-located CST axons showed no decussation (Fig. 1E, F). Consequently, the area of PKCγ-stained CST in the spinal dorsal funiculus was much decreased, while a small proportion of CST axons appeared in the lateral funiculus, which was not observed in control mice (Fig. 1G–J).

To further confirm the CST phenotype, we administered a unilateral BDA injection into the motor cortex to show the CST trajectory. BDA-labeled CST axons in the ventral hindbrain split into two bundles, and the axons in the medial bundle crossed the midline and entered the contralateral dorsal funiculus, whereas those in the lateral bundle failed to do so in Ntn1^Gfap^ CKO mice (Fig. 1K–P). The uncrossed CST axons eventually entered the lateral funiculus on the ipsilateral side (Fig. 1Q–T). Thus, Netrin-1 deficiency leads to defasciculation of CST axons in the hindbrain and a failure of midline crossing at the caudal medulla, resulting in an ipsilateral descending spinal lateral funiculus.

In addition, the cellular architecture of the cerebral cortex was also examined to exclude the possibility that abnormal cell arrangement in the cortex contributes to malformations of the CST in Ntn1^Gfap^ CKO mice. Nissl staining showed that the cortical layers were well-maintained in CKO mice in comparison with those in the controls (Fig. S2A, B). CST neurons were located in layer V, and the distribution of pyramidal neurons in layer V shown by *PlxnD1*, *DKK3* and *Ctip2* displayed no obvious differences between CKO and control mice (Fig. S2C–H).

### Cortical Netrin-1 is not involved in the midline crossing of the CST axons

*Netrin-1* is also expressed in the developing cerebral cortex, where CST neurons are located [27], and *Netrin-1* was inactivated in the cortex of Ntn1^Gfap^ CKO mice (Fig. 1A). To investigate whether cortical Netrin-1 contributed to the CST phenotype, we generated Ntn1^Emx1^ CKO mice. Emx1-driven Cre was present in cortical pyramidal neurons, including CST neurons in layer V, and Cre expression was initiated on embryonic day (E) 11.5 [28], ensuring the deletion of Netrin-1 in cortical neurons before the initiation of CST development. Netrin-1 deletion in the cerebral cortex was confirmed by western blot, whereas its expression in the cerebellum was unaffected (Fig. S3I). In the analysis of the CST by PKCγ immunohistochemistry, no detectable differences were observed in the CST trajectory between Ntn1^Emx1^ CKO and control mice (Fig. S3A–H). AuCl_3_ staining showed similar results (Fig. S3E, F). These results demonstrated that Netrin-1 expression in the cerebral cortex is not involved in CST development.

### Netrin-1 expression in the ventral VZ of hindbrain is reduced in CKO mice

**Fig. 2:**
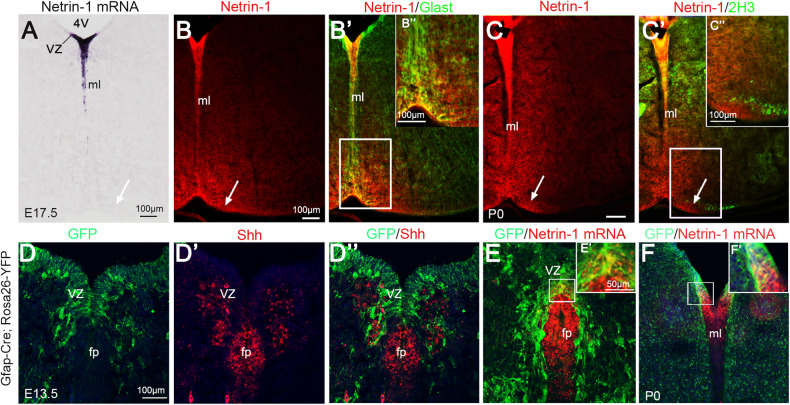
Netrin-1 protein in the ventral medulla is transported by the process of Glast-positive radial glia cells in the VZ of hindbrain. **A** *Netrin-1* mRNA is present in the VZ and dorsal midline region of hindbrain at E17.5 (**A**). **B**-B” Netrin-1 protein is not only present in the regions containing *Netrin-1* mRNA, but also in the ventral hindbrain (arrow), where contains no mRNA at E17.5 (arrow in **A**). Double immunostaining of Netrin-1 (red) and Glast (green) shows the localization of Netrin-1 in Glast-positive process of radial glia cells in the ventral medulla. **B**-B’ are the same filed, respectively. Boxed area in (B’) is enlarged in (B”). **C**-C” Double immunostaining of Netrin-1 (red) and 2H3 (green) shows overlapping of 2H3-positve CST axons and Netrin-1 protein in the ventral medulla at P0. Boxed area in (C’) is enlarged in (C”). **D**-D” The GFP signal is widely detected in the VZ of the hindbrain at E13.5 but excluded by the floor plate labeled by *Shh*. **E**, **F** The Netrin-1 expression in the VZ is overlapped with GFP signal at E13.5 (**E**) and P0 (**F**). Boxed area in **E** and **F** is enlarged in (E’) and (F’), respectively. The representative images were taken in the middle level of medulla oblongata where pyramidal tract accumulated in the ventral margin. Scale bars = 50 μm (E’, F’) and = 100 μm in other panels. 4 V 4th ventricle, VZ ventricular zone, fp floor plate, ml midline.

Intense *Netrin-1* mRNA signals were detected in the ventral VZ and dorsal half of the midline region in control mice at E17.5, the latter correspond to residual floor plate (Fig. 2A). Although *Netrin-1* mRNA and protein overlapped in these two regions, Netrin-1 protein was also detected in the ventral medulla around the midline, which contained no *Netrin-1* mRNA (arrow, Fig. 2B). Previous studies have shown that Netrin-1 produced by neural progenitors is transported by their processes to the pial surface to form a growth substrate in the spinal cord and corpus callosum [29, 30]. To investigate whether this was the case in the hindbrain, we conducted double staining of Netrin-1 and the radial glia cell marker, Glast, and found that the Netrin-1 protein was colocalized in Glast^+^ processes extending from the VZ to the ventral medulla (Fig. 2B’-B”). Importantly, we also found that Netrin-1 protein was present in the vicinity of primitive CST axons labeled with 2H3 antibodies (arrow, Fig. 2C-C”). These results suggested that Netrin-1 protein might be transported by radial glial cells to the ventral medulla, through which CST axons pass.

**Fig. 3:**
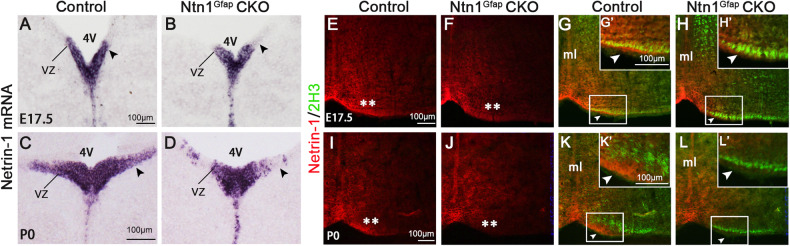
The deletion of *Netrin-1* mRNA in the VZ and reduction of Netrin-1 protein in the ventral margin of Ntn1^Gfap^ CKO mice. **A**–**D** In situ hybridization shows that *Netrin-1* transcripts are dramatically decreased in the VZ of Ntn1^Gfap^ CKO mice at E17.5 (**A**, **B**) and P0 (**C**, **D**) compared with controls. **E**–**L** Double staining of Netrin-1 (red) and 2H3 (green) shows that Netrin-1 protein in the ventral margin (asterisks) is located in the proximity of 2H3-labeled CST axons (triangles) of control mice, but it is obviously decreased in CKO mice at E17.5 (**E**–**H**) and P0 (**I**–**L**). **E**–**H** are the same fields, respectively. Scale bars = 100 μm. 4 V, 4th ventricle. **I**–**L** are the same fields, respectively. The inserts in **G**, **H**, **K**, **L** are enlarged boxed areas in (G’, H’, K’, L’). The representative images were taken at the caudal level of medulla oblongata where pyramidal tract accumulated in the ventral margin. This experiment was replicated in three different control and CKO mice. Scale bars = 100 μm. 4 V 4th ventricle, ml midline, VZ ventricular zone.

The extension of CST axons reaches the caudal medulla before birth, thus, the split CST axons in adult Ntn1^Gfap^ CKO mice are very likely to occur during the embryonic stages. Therefore, a specific riboprobe for in situ hybridization targeting the sequence of exon 2 spanning two loxp sites was prepared, and then mRNA and protein distributions were compared between Ntn1^Gfap^ CKO and control at embryonic stages. The results showed that the distribution of *Netrin-1* mRNA in the midline region was not altered (Fig. S4), but *Netrin-1* mRNA in the VZ, particularly in the lateral portion, was reduced at E17.5 and P0 (black arrowheads, Fig. 3A–D). Interestingly, in addition to the VZ, the region near the CST in the ventral hindbrain also showed a reduced intensity of Netrin-1 immunofluorescence (asterisks, Fig. 3E, F, I, J). Considering the role of Netrin-1 in axonal fasciculation during embryonic development [31, 32], the reduction in Netrin-1 protein transported from the VZ to the ventral hindbrain is likely contributing to the defasciculation of CST axons and consequently, laterally-located bundles of CST axons extended their processes ipsilaterally into the lateral funiculus of the spinal cord in Ntn1^Gfap^ CKO mice.

A recent study showed that deletion of floor plate-derived *Netrin-1* by Shh-Cre also affects the decussation of CST axons [17]. Gfap-driven Cre recombinase is active in the radial glia cells in the VZ but not in the floor plate [18]. To confirm this, we generated the Gfap-cre: Rosa26-YFP mice in which GFP signal represents Cre activity. The results showed that GFP-positive cells were detected in the VZ but not in the floor plate at E13.5 (Fig. 2D), and this was further confirmed by double staining with *Shh*, a specific marker for the floor plate, showing that no GFP-positive-cells contained *Shh* mRNA (Fig. 2D-D”). Noteworthy, the *Netrin-1* mRNA signals were colocalized with GFP-positive cells in the VZ at both E13.5 and P0 (Fig. 2E, F). Thus, the CST phenotype in Ntn1^Gfap^ CKO mice is caused by inactivation of *Netrin-1* in the VZ of hindbrain rather than in the floor plate.

### Ntn1^Gfap^ CKO mice display asymmetric movements

**Fig. 4:**
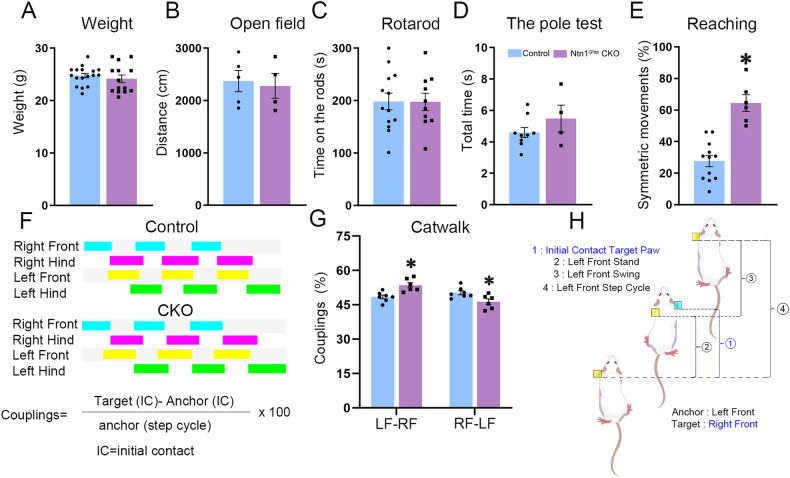
Alterations of locomotor-related behaviors in the Ntn1^Gfap^ CKO mice. **A** The body weight is comparable between control and CKO mice. By Shapiro–Wilk test, the samples followed a normal distribution. Student’s *t* test, *p* > 0.05, *N = 17 for control and *N* = 14 CKO mice. **B**, **D** No significant differences are found between the two groups in the traveled distance in the open-field (**B**), the time on the rods in the rotarod test (**C**) and the total time for mice landed to the floor in the pole test (**D**). **E** Ntn1^Gfap^ CKO mice display more symmetric forelimb movements than the controls in the exploratory reaching test. By Shapiro–Wilk test, the samples followed a normal distribution. Student’s *t* test, *p* < 0.05, *, *N* = 12 for control and *N* = 6 for CKO mice. **F**–**H** In the catwalk test, couplings describe the temporal relationship of two paws. The homologous coupling limb pair LF-RF is increased, whereas the values for limb pairs RF-LF were decreased in Ntn1^Gfap^ CKO mice compared with controls. By Shapiro–Wilk test, the samples followed a normal distribution. Student’s *t* test, *p* < 0.05, *, *N* = 7 for control and *N* = 6 for CKO mice. **F** Footprints of control and CKO mice and calculations (expressed as %) of coupling parameters. **H** Schematic representation for the pair LF-RF, in which LF acts as an Anchor Paw (yellow) and RF as a Target paw (blue).

We conducted behavioral tests to explore whether a defective CST is involved in the pathogenesis of mirror movements. The CKO mice and age-matched control mice showed no significant differences in the body weight, total distance traveled in the open-field test, duration of the rods in the rotarod test or total time in the pole test (Fig. 4A–D).

The exploratory reaching test is widely used to evaluate the lateralization of voluntary forelimb movements [33]. Mice tended to establish contact with the walls using their forelimbs in an asymmetric or symmetric manner when placed in a novel glass cylinder. Ntn1^Gfap^ CKO mice showed more symmetric forelimb movements than controls (Student’s *t* test, *p* < 0.001; Fig. 4E). In addition, we examined gait parameters using the catwalk test and found that couplings, which reflect the temporal relationship between the placement of two paws within a step cycle were impaired (Fig. 4F–H). The homologous coupling for the limb pair LF-RF (left forepaw-right forepaw) was increased (Student’s *t* test, *p* < 0.05; Fig. 4F), while that for RF-LF showed a decreased trend in Ntn1^Gfap^ CKO mice compared with control group (Student’s *t* test, *p* = 0.09; Fig. 4F), indicating that right forepaw was delayed during swing while the left forepaw was in stance in CKO mice, reflecting the symmetric movements in the catwalk assay. Other gait parameters including stride length, max intensity, swing duration, duty cycle, swing speed and paw areas did not differ between the two groups in any of the four paws (Fig. S5A–F). We also conducted Aucl3 staining and Nissl staining to examine the organization of spinal cord and no obvious defects were observed in CKO mice (Fig. S5G, H). Overall, these findings revealed that deletion of Netrin-1 expression in the VZ of hindbrain impairs the production of voluntary asymmetric movements.

## Discussion

**Fig. 5:**
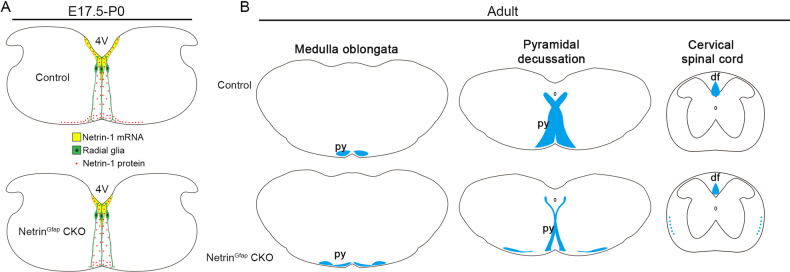
The summary of the CST phenotypes in Ntn1^Gfap^ CKO mice. **A** In embryonic hindbrain, *Netrin-1* mRNA in the ventricular zone (VZ) is reduced in CKO mice, and accordingly, radial glial cell (green)-transported Netrin-1 protein (red dots) in the proximity of CST axons is decreased. **B** In adult CKO mice, the CST axons (blue) split into two bundles in the ventral hindbrain. The medial one crosses the midline, whereas the lateral one fails to do so and enters into the ipsilateral lateral funiculus of the spinal cord. 4 V 4th ventricle, df dorsal funiculus, ml midline, py pyramidal tract.

Voluntary movement on one side of the body is controlled by the contralateral cortex via the CST, the axons of which cross the midline at the caudal medulla (pyramidal decussation) for contralateral innervation. CMM involves malformations of pyramidal decussation, in which some CST axons do not cross the midline, resulting in bilateral innervation and consequent mirror movement. A previous study has shown that inherited mutations in Netrin-1 are present in patients with CMM, and both crossed and uncrossed CST are present in the cervical spinal cord [12]. Here, we show that the deletion of Netrin-1 in the VZ of hindbrain leads to defasciculation of the CST in the ventral hindbrain and laterally-located CST axons descending ipsilaterally in the spinal cord without midline crossing (Fig. 5). Behavioral examinations reveal increased symmetric movements in Ntn1^Gfap^ CKO mice. Taken together, these results indicate that Netrin-1 is a genetic factor involved in the pathogenesis of CMM.

Netrin-1 and its receptors (e.g., DCC and Unc5c) play essential roles in the pathfinding of commissural axons during embryonic development, as evidenced by the loss of the corpus callosum and the failure of midline crossing of spinal commissural axons in knockout mice [10, 30, 34]. Conventional Netrin-1 knockout mice die after birth; however, CST development, particularly the growth of CST axons within the spinal cord, continues after birth [15]. In this study, Ntn1^Gfap^ CKO mice are generated and survived until adulthood without gross abnormalities. We show that some uncrossed CST axons are ectopically located in the lateral funiculus of the spinal cord in Ntn1^Gfap^ CKO mice. Similar phenotypes have been reported in Netrin-1 receptor knockout mice, in which both crossed and uncrossed CST axons are present in the Unc5c mutant and DCC Kanga mice carrying a spontaneous viable mutation in DCC [14, 15] as well as Ntn1^Shh^ CKO mice [17]. These results provide novel evidence supporting the idea that the components of the Netrin-1 signalling pathway are implicated in the pathfinding of CST axons during embryonic and postnatal development, and the disruption of this pathway is one of the key factors involved in the onset of CMM.

Netrin-1 is widely expressed in the brain, including the cerebral cortex, where CST neurons are located. To explore if cortical Netrin-1 is involved in defective CST development, we selectively inactivate Netrin-1 in cortical pyramidal neurons by generating Ntn1^Emx1^ CKO mice, in which CST axons are well-maintained, hinting that cortical Netrin-1 might not be essential for CST development. We also noted that the trajectory of the CST above the level of the hindbrain is not obviously altered in Ntn1^Gfap^ CKO mice, and that the appearance of CST abnormalities is first detected in the medulla oblongata along the long descending route from the cortex to the spinal cord. Thus, Netrin-1 expression in the hindbrain, but not in the forebrain or midbrain, is likely to be required for CST development.

The role of Netrin-1 in the spinal cord has been studied extensively. Several recent studies have argued against the classic view that Netrin-1 acts as a diffusible chemoattractant to direct the ventral growth of spinal commissural axons via a floor plate-derived Netrin-1 gradient [29, 35], and it has been proposed that VZ-derived Netrin-1 accumulates on the pial surface adjacent to the path of commissural axon extension, which promotes the anchoring of pioneer commissural axons close to the pial surface in spinal cord [29, 35]. In our study, floor plate-derived Netrin-1 was not targeted by Cre activity, and the reduction of Netrin-1 transcript took place only in the VZ of the hindbrain. We speculated that the VZ-derived Netrin-1 protein is transported to the ventral margin and accumulates near the developing CST axons, and the reduction may lead to the defasciculation of CST axons and then split into two bundles, consistent with the finding in the spinal cord that VZ-derived Netrin-1 might influence commissural axon fasciculation. The laterally-located bundle fails to respond correctly to other cues located at the midline and descends ipsilaterally into the spinal cord, leading to bilateral innervation by one side of the cerebral cortex. Notably, a recent study revealed that floor plate-produced Netrin-1 in the hindbrain is also involved in the midline crossing of CST axons. In the absence of Netrin-1 in the floor plate, the CST spreads laterally, and fewer CST axons are detected in the spinal dorsal funiculus, and the other CST axons are observed in the ventral and lateral funiculus of the spinal cord [17], which is more severe than the phenotypes observed in the Ntn1^Gfap^ CKO mice. Combining the present findings from Ntn1^Gfap^ CKO and those from Ntn1^Shh^ CKO mice, both VZ- and floor plate-derived Netrin-1 might play a complementary role in the CST axon guidance.

The role of Netrin-1 in axonal fasciculation during embryonic development is well-documented. Netrin-1 belongs to the laminin superfamily, which most closely resembles the laminin γ chain [8, 36], making it plausible that Netrin-1 may influence adhesion between axons. Previous studies have demonstrated that Netrin-1 is involved in cell adhesion in other systems [37, 38], and recent investigations have shown that Netrin-1 and its receptor DCC are associated with Draxin to mediate axon guidance and fasciculation [32, 39]. Abnormal decussations of the CST have also been reported in Sema6A and NCAM mutants [40, 41]. Further studies are needed to explore whether Draxin is involved in Netrin-1-implicated formation of the CST, and whether Sema6A and NCAM interact with Netrin-1 and DCC in the pathfinding of the CST.

We also explore the behavioral consequences of aberrant CST projections in the Ntn1^Gfap^ CKO mice. Ntn1^Gfap^ CKO mice exhibited specific motor impairments, as shown by the increase of voluntary symmetric forelimb movements in exploratory reaching behaviors and impaired homologous coupling in catwalk test. Notably, they do not exhibit a hopping gait like DCC Kanga mice. Conditional deletion of the DCC in the spinal cord has been demonstrated to be sufficient to cause the hopping gait observed in DCC Kanga mice. Thus, the neuronal circuits responsible for hopping gait might rely on spinal commissural circuits rather than on proper CST wiring [24, 33, 42]. It should be noted that unknown defects may be present in Ntn1^Gfap^ CKO mice, particularly in brain regions associated with motor control, and thus, the motor impairments cannot simply be attributed to the defective formation of the CST.

In summary, our study demonstrated that VZ-derived Netrin-1 in the hindbrain is required for fasciculation and decussation of the CST axons. The increased voluntary symmetric forelimb movements in Ntn1^Gfap^ CKO mice add novel evidence for the involvement of the Netrin-1 signalling pathway in the pathogenesis of CMM.

### Supplementary information

original data

Supplementary data

## Data Availability

All data generated in this study are included either in this article or in the Supplementary Information files.
