# Supplementary material for: Ventricular Netrin-1 deficiency leads to defective pyramidal decussation and mirror movement in mice
Source: Cell Death Dis. 2024 May 17;15(5):343. doi: 10.1038/s41419-024-06719-1 (PMC11101614; doi:10.1038/s41419-024-06719-1)
Supplement: Supplementary file 1 — original data [file 41419_2024_6719_MOESM1_ESM.docx]

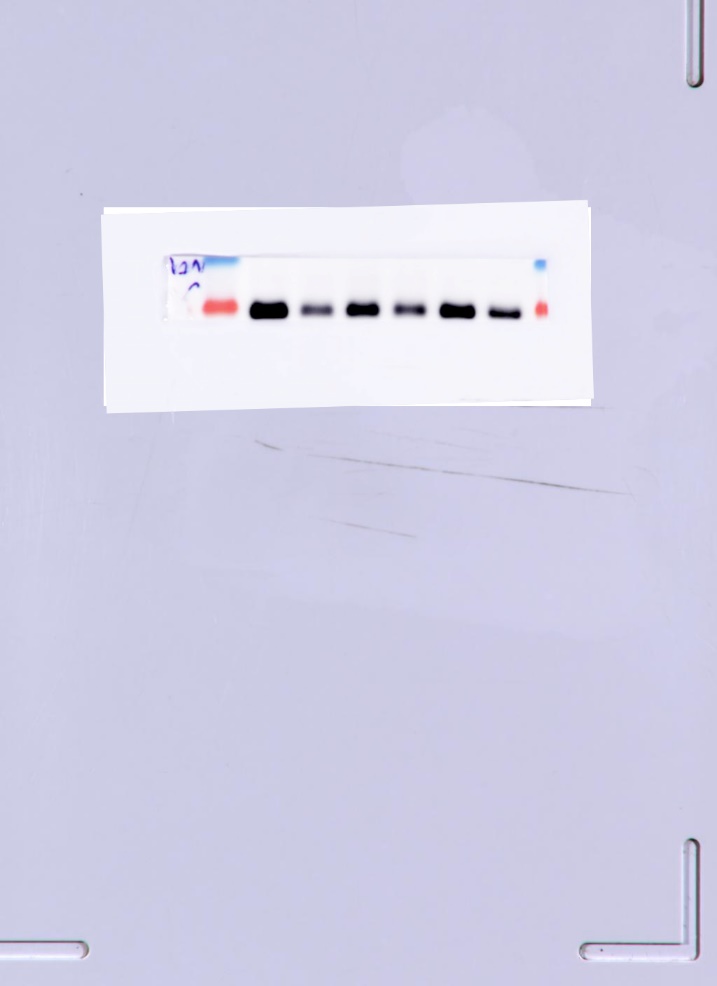


anti-netrin 1:1000 abcam

anti-GAPDH 1:1000 epizyme


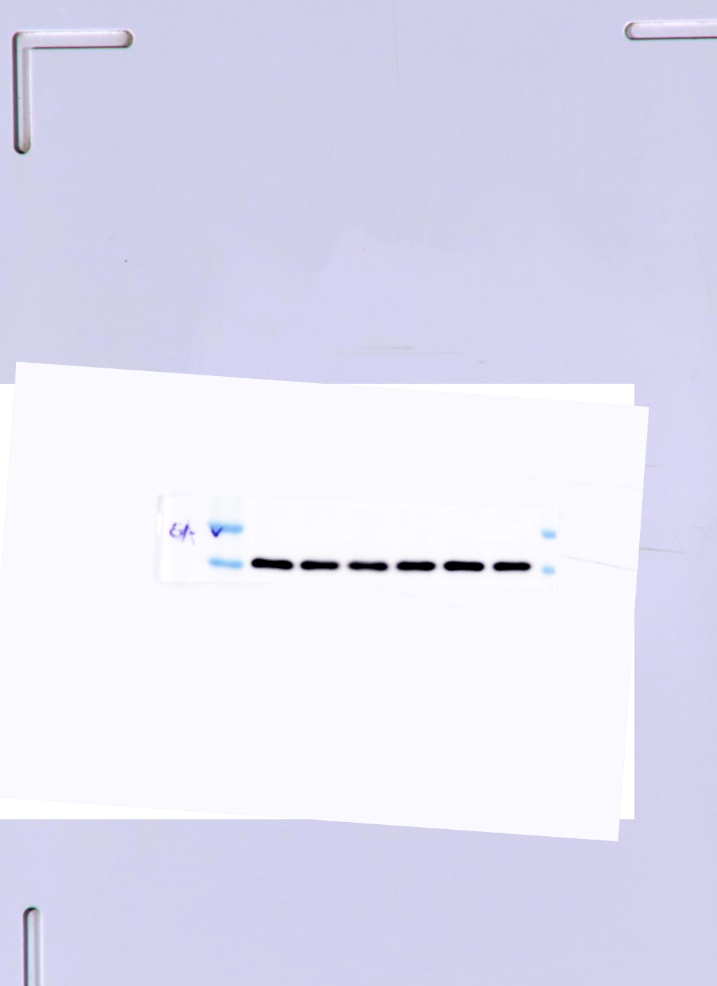


上样量自左向右分别是：control-1-ctx; Ntn1^Gfap^ CKO-1-ctx; control-2-ctx; Ntn1^Gfap^ CKO-2-ctx; control-3-ctx; Ntn1^Gfap^ CKO-3-ctx;

anti-netrin 1:1000 abcam


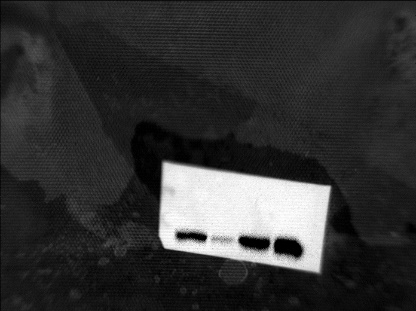


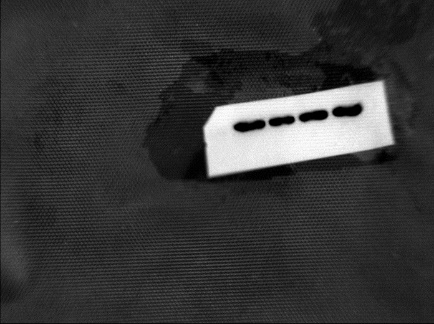


上样量自左向右分别是：control-ctx; Ntn1^Emx1^ CKO-ctx; control-cb; Ntn1^Emx1^ CKO-cb;

anti-GAPDH
