# Supplementary material for: Ventricular Netrin-1 deficiency leads to defective pyramidal decussation and mirror movement in mice
Source: Cell Death Dis. 2024 May 17;15(5):343. doi: 10.1038/s41419-024-06719-1 (PMC11101614; doi:10.1038/s41419-024-06719-1)
Supplement: Supplementary file 2 — Supplementary data [file 41419_2024_6719_MOESM2_ESM.pdf]

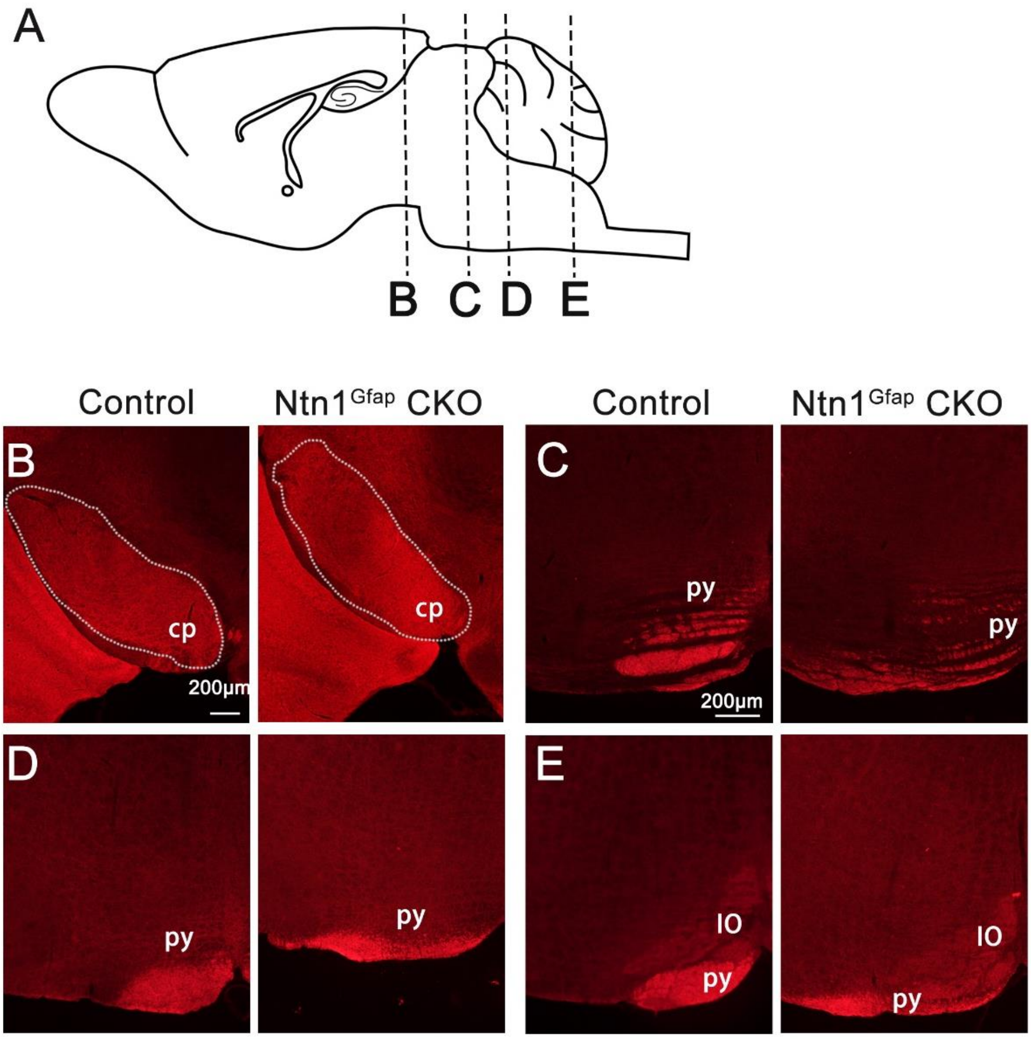

Supplementary Figure 1. The morphology of CST was not obviously altered in Ntn1^{Gfap} CKO mice at the level of cerebral peduncle. (A) Schematic representation of a sagittal section indicating the trajectory of the CST and the level of the coronal sections presented in this study. (B) Staining with anti-PKCγ antibody shows no significant difference at the level of cerebral peduncle between the two genotypes (B). (C-E) In the route to the pyramidal decussation, PKCγ-stained pyramidal tract was evident in the ventral hindbrain of control mice at the levels of the pons (C), rostral medulla (D) and caudal medulla (E), but it broadened in the mediolateral direction and spread into two bundles in the CKO mice: the medial bundle was maintained in the normal position but the other one was located laterally. Scale bars = 200 μm. cp, cerebral peduncle. py, pyramidal tract. IO, inferior olive.

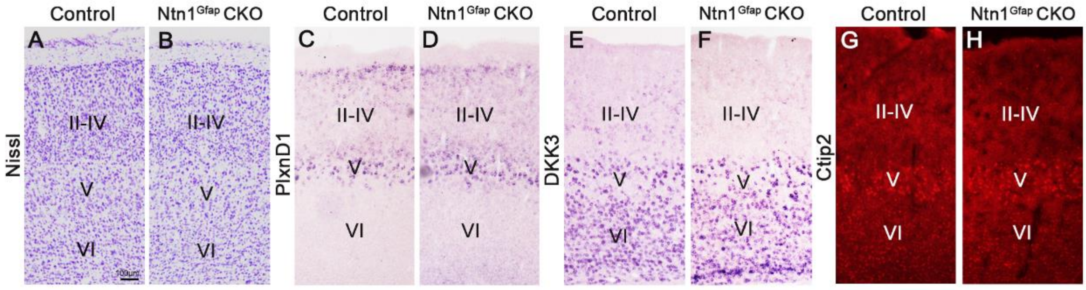

Supplementary Figure 2. Layered architecture is well maintained in $Ntn1^{Gfap}$ CKO mice. (A, B) Nissl staining shows cortical layers are comparable between control and CKO mice. (C-H) The distributions of layer V-specific markers, *PlxnD1* (C, D), *DKK3* (E, F) and *Ctip2* (G, H) show no obvious differences between control (C, E, G) and CKO mice (D, F, H). II-VI, cortical layers. Scale bar = 100 μm in (A), applies to (B-H).

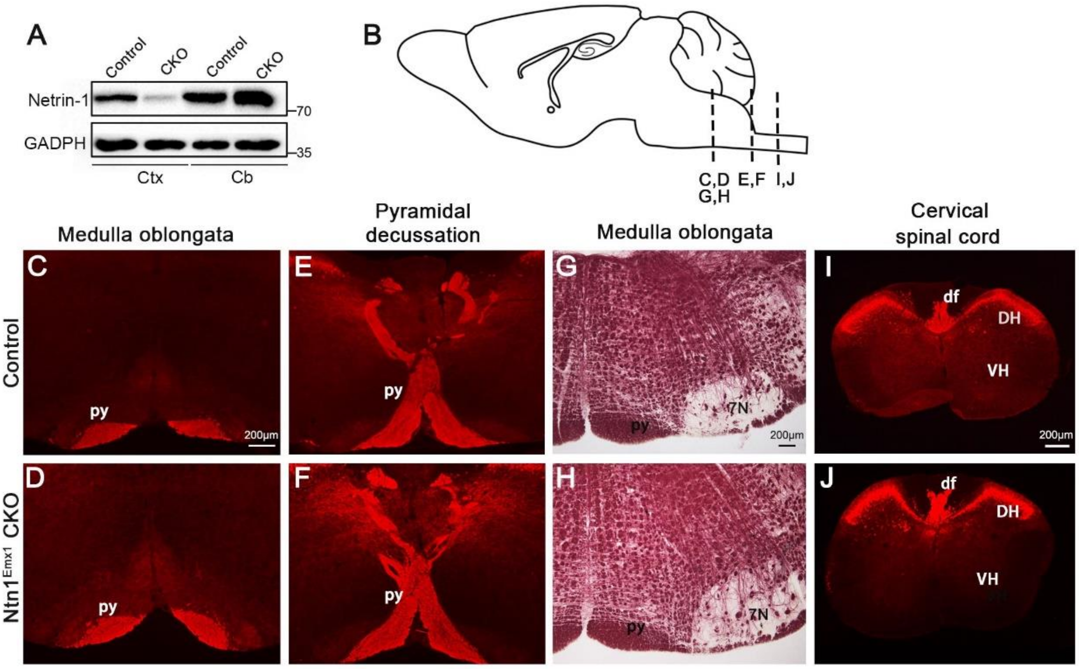

Supplementary Figure 3. The CST morphology is intact in Ntn1^{Emx1} CKO mice. (A) Western blot data shows that Netrin-1 expression is decreased in the cortex but not in the cerebellum of Ntn1^{Emx1} CKO mice. (B) Schematic representation of a sagittal section indicating the trajectory of the CST and the level of the coronal sections presented in this study. (C-J) PKCγ staining (red) shows the location of pyramidal tract in ventral hindbrain (C, D), and pyramidal decussation at the level of caudal medulla (E, F) in control (C, E) and Ntn1^{Emx1} CKO mice (D, F). (G, H) Aucl3 staining shows that the location of pyramidal tract in the ventral medulla of control (G) and Ntn1^{Emx1} CKO mice (H). (I, J) PKCγ-stained CST axons in the dorsal funiculus of cervical spinal cord of control (I) and Ntn1^{Emx1} CKO mice (J). Scale bars = 200 μm. 7N, facial nucleus; Cb, cerebellum; Ctx, cerebral cortex; df, dorsal funiculus; DH, dorsal horn; VH, ventral horn; py, pyramidal tract.

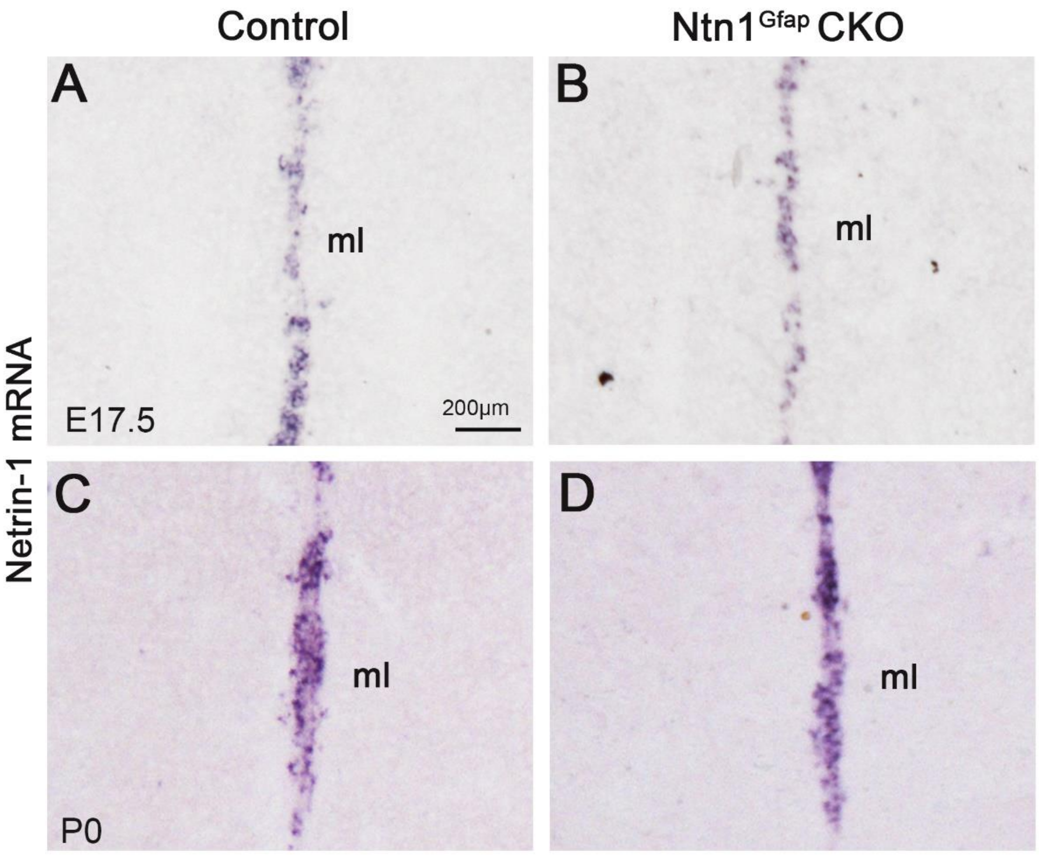

Supplementary Figure 4. The expression of Netrin-1 was similar between control and Ntn1^{Gfap} CKO mice in the midline. (A-D) *In situ* hybridization shows that Netrin-1 transcripts were comparable in Ntn1^{Gfap} CKO mice at E17.5 (A, B) and P0 (C, D) compared with controls. Scale bars = 200 μm. ml, midline.

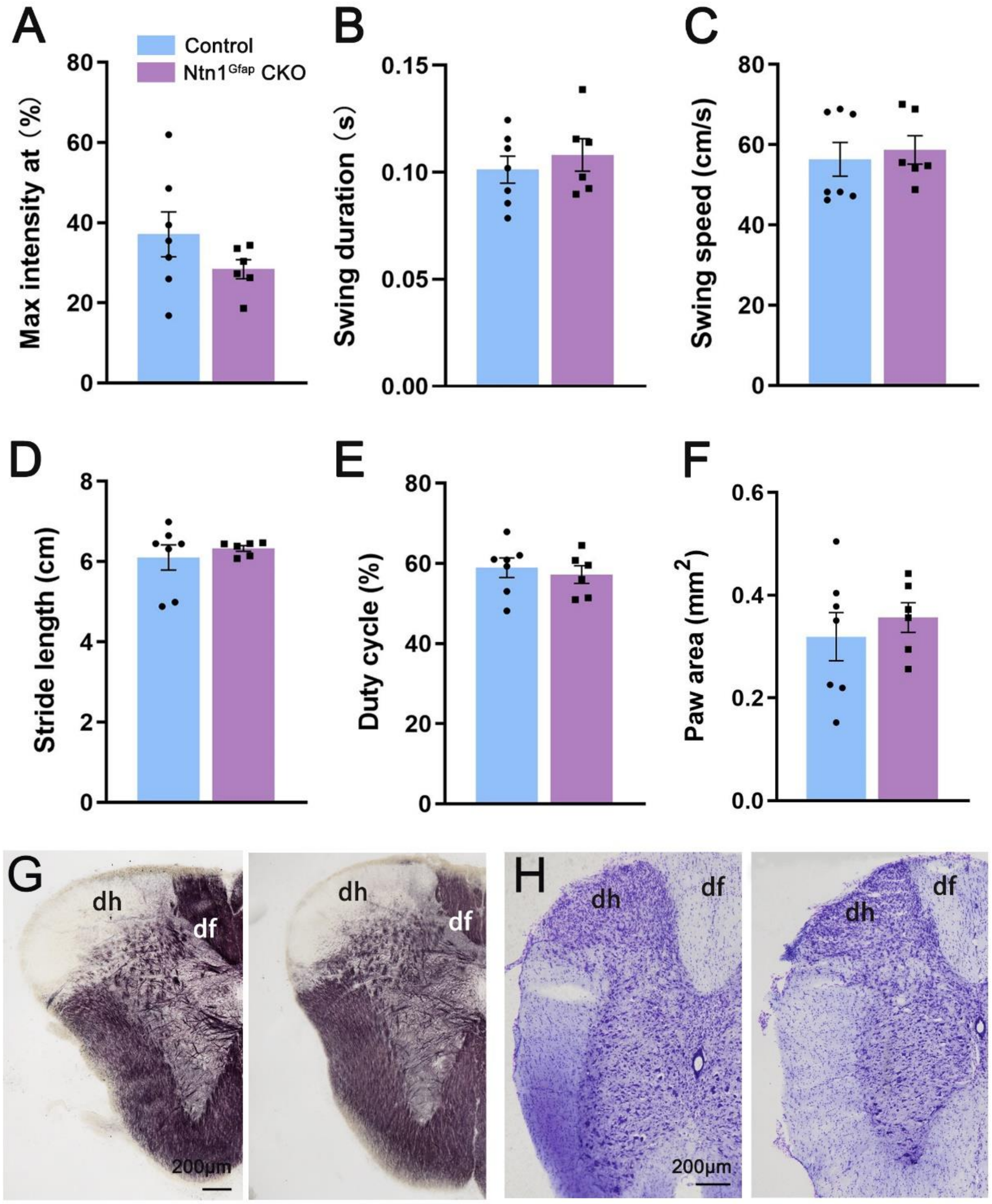

Supplementary Figure 5. No obvious differences were observed in the gait parameters and organization of spinal cord in Ntn1^{Gfap} CKO mice. (A-F) There were no significant difference in the max intensity (A, p=0.20), swing duration (B, p=0.50), swing speed (C, p=0.68), stride length (D, p=0.52), duty cycle (E, p=0.62) and paw areas (F, p=0.53) between control and CKO mice. Student's *t* test. N=7 for control and N=6 for Ntn1^{Gfap} CKO mice. (G-H) Aucl3 staining and Nissl staining were performed in control and Ntn1^{Gfap} CKO mice and no obvious defects were observed. Scale bars = 200 μm, dh,

dorsal horn, df, dorsal funiculus.
